# Tetrameric Structures of Inorganic CBS-Pyrophosphatases from Various Bacterial Species Revealed by Small-Angle X-ray Scattering in Solution

**DOI:** 10.3390/biom10040564

**Published:** 2020-04-07

**Authors:** Liubov A. Dadinova, Ekaterina Yu. Soshinskaia, Cy M. Jeffries, Dmitri I. Svergun, Eleonora V. Shtykova

**Affiliations:** 1 Shubnikov Institute of Crystallography of Federal Scientific Research Centre “Crystallography and Photonics” of Russian Academy of Sciences, Leninskiy prospect, 59, 119333 Moscow, Russia; katuffus@rambler.ru (E.Y.S.); eleonora.shtykova@gmail.com (E.V.S.); 2 EMBL, Hamburg Unit, c/o DESY, Notkestr. 85, Geb. 25a, 22607 Hamburg, Germany; cy.jeffries@embl-hamburg.de (C.M.J.); svergun@embl-hamburg.de (D.I.S.)

**Keywords:** inorganic pyrophosphatase, cystathionine-β-synthase, CBS-domain, CBS-pyrophosphatases, size-exclusion chromatography, multi-angle laser light, structural modeling

## Abstract

Quaternary structure of CBS-pyrophosphatases (CBS-PPases), which belong to the PPases of family II, plays an important role in their function ensuring cooperative behavior of the enzymes. Despite an intensive research, high resolution structures of the full-length CBS-PPases are not yet available making it difficult to determine the signal transmission path from the regulatory to the active center. In the present work, small-angle X-ray scattering (SAXS) combined with size-exclusion chromatography was applied to determine the solution structures of the full-length wild-type CBS-PPases from three different bacterial species. Previously, in the absence of an experimentally determined full-length CBS-PPase structure, a homodimeric model of the enzyme based on known crystal structures of the CBS domain and family II PPase without this domain has been proposed. Our SAXS analyses demonstrate, for the first time, the existence of stable tetramers in solution for all studied CBS-PPases from different sources. Our findings show that further studies are required to establish the functional properties of these enzymes. This is important not only to enhance our understanding of the relation between CBS-PPases structure and function under normal conditions but also because some human pathogens harbor this class of enzymes.

## 1. Introduction

Inorganic pyrophosphatases (PPases) are enzymes present in all living organisms. PPases catalyze hydrolysis of inorganic pyrophosphate into two molecules of orthophosphate, providing the necessary thermodynamic conditions for important reactions like protein-, RNA-, and DNA synthesis, and making these enzymes essential for life [1].

Soluble PPases belong to three different non-homologous families: I, II, and III. Family II PPases were discovered in 1998 [2,3] and are still intensively investigated. These enzymes exist almost exclusively in bacteria and archaebacteria, including human pathogens. Generally, family II pyrophosphatases are composed of two well-defined domains connected by a flexible linker, N-terminal DHH and C-terminal DHHA2, and they belong to the DHH (Asp-His-His) family of phosphohydrolases [4]. The flexible linker between the N- and C-terminal domains and the active sites are located at the domain interfaces [5,6]. The C-terminal domain contains a high-affinity substrate-binding site, whereas the catalytic site that binds the nucleophile-coordinating metal cations is located at the N-terminal domain. Importantly, substrate binding to the C-terminal domain in its open conformation causes the domain closure [7].

About one-quarter of family II PPase enzymes contains a 250-residue insertion within the N-terminal domain [8]. The insertion consists of the DRTGG domain, named after its conserved Asp-Arg and Thr-Gly-Gly motifs (about 120 amino acids)), and two so-called CBS domains, named after cystathionine-β-synthase, where they were identified for the first time. These PPases are called CBS-PPases to distinguish them from common family II PPases. CBS domains bind adenine nucleotides with various affinities, acting as sensors of the cellular energy status [9].

Quaternary structure of CBS-pyrophosphatases plays an important role in their activity and contributes to the thermal stability of these enzymes. It is known that the PPases are more active in the dimeric form with four CBS domains than in the monomeric state, exhibiting positive kinetic cooperativity, which is lost upon CBS domains removal [10,11]. The CBS domains, in turn, contribute to aggregation of the PPases in solution. That is why the crystal structure of human cystathionine-β-synthase was only solved for the protein variant lacking the CBS domains [12]. This protein was found to be dimeric in the crystal and it was speculated that the CBS pair may prompt tetramerization of the full-length protein in solution [13], however, no experimental evidence for the tetramerization has been obtained so far. It is worth emphasizing that, despite fairly intensive structural studies of the family II PPases, only a few high resolution three-dimensional structures of family II PPases are available [5,6,14].

The high resolution structure of the full-length CBS-PPase has not yet been obtained, and only limited information is available to understand both the main regulatory mechanisms of interaction between domains and the signal transmission paths between catalytic and regulatory domains. It is known that the DRTGG domain is important for the linear diadenosine polyphosphates ApnA binding [15]. However, the available data is not sufficient to establish the regulation mechanism of the enzyme and to determine the signal transmission path from the regulatory to the active center. These questions cannot be resolved without elucidating the structure of the full-length enzyme, and structural analysis of the full-length CBS-PPase in native conditions remains to be of primary importance. Small-angle X-ray scattering (SAXS) was chosen in the present work as a main research approach, since it can be used to structurally characterize biological macromolecules directly in solution, i.e., at close to the physiological conditions [16]. It is highly likely that CBS-PPase may exist in solution as mixtures of various oligomeric forms, and we utilized a size exclusion chromatography (SEC) in combination with SAXS to produce and analyze CBS-PPases in their monodisperse states. The SEC-SAXS with multi-angle laser light scattering combines the advantages of a purification and an analytical method allowing one to characterize oligomeric states of individual proteins and to analyze the formation/dissociation of complexes [17].

We present here a structural study of CBS-PPases from *D. hafniense* (dh-PPase), *E. lenta* (el-PPase), and *E. harbinense* (eh-PPase) by SAXS with on-line SEC-SAXS with multi-angle laser light scattering yielding information on the domain organization, quaternary structure and oligomeric state of these proteins in solution. The obtained structural results should further help in elucidating the functional peculiarities of the CBS-PPases.

## 2. Materials and Methods

### 2.1. Sample Preparation

Bacterial inorganic pyrophosphatases dh-PPase, its truncated variant without regulatory part ΔCDC-dh-PPase, eh-PPase, and el-PPase were expressed and purified by Viktor Anashkin’s group from Belozersky Institute of Physico-Chemical Biology, Lomonosov Moscow State University following procedures that have been reported elsewhere [11]. The samples are from the same batch as those already published in [18], and the relevant SDS-PAGE is presented in Figure 2 of that paper. Further control of monodispersity was done by utilizing the on-line SEC coupled with SAXS.

The family II PPases are Co^2+^ or Mn^2+^ metalloenzymes that require Mg^2+^ for catalysis and the activities of CBS-PPases preincubated with 0.1 mM Co^2+^ are higher than those incubated with Mn^2+.^ Therefore, Co^2+^ was routinely used as the transition metal cofactor. Besides, Co^2+^ was also added to stabilize the enzymes [8]. The specimens were resuspended into the buffer containing 0.1 M MOPS-KOH buffer, pH 7.2, 2 mM MgCl_2_, 0.1 mM CoCl_2_, and 150 mM KCl and utilized for SAXS and SEC-SAXS without further modification.

Protein concentrations were determined spectrophotometrically, using A^0.1%^_280_ value of 0.477 and 0.419 for the wild type and truncated proteins, respectively, as calculated from the amino acid composition with ProtParam [19]. Molar concentrations were calculated on the basis of the subunit molecular mass of 33.73 kDa (ΔCDC-dh-PPase), 60.35 kDa (dh-PPase), 47.68 kDa (eh-PPase), and 49.28 kDa (el-PPase). The concentration during proteins isolation was 1 mg/mL (dh-PPase), 0.45 mg/mL (ΔCDC-dh-PPase), 1.1 mg/mL (el-PPase), and 2.1 mg/mL (eh-PPase).

### 2.2. Scattering Experiments and Data Analysis

Synchrotron SAXS measurements were performed at the European Molecular Biology Laboratory (EMBL) on the EMBL-P12 BioSAXS beam line at the PETRAIII storage ring (DESY, Hamburg) [19] equipped with a robotic sample changer and a 2D photon counting pixel X-ray detector Pilatus 2M (DECTRIS, Switzerland) at a sample to detector distance of 3.1 m with a sample path length of 1.5 mm. The scattering intensity, I(s), was recorded in the range of the momentum transfer 0.027 < s < 4.0 nm^−1^, where s = (4πsinθ)/λ, 2θ is the scattering angle, and λ = 0.124 nm, the X-ray wavelength [20]. The measurements were carried out at 10 °C using continuous sample flow operation over a total exposure time of 1 s, collected as 20 × 50 millisecond individual frames to monitor for potential radiation damage (no radiation effects were detected [21]). The data were corrected for the solvent scattering and processed using standard procedures [22].

To account for interparticle interactions, we measured and compared samples at different concentrations between 2–10 mg/mL before SEC-SAXS procedure. No concentration dependence was observed (Figure S1).

The on-line size-exclusion chromatography (SEC-SAXS) with multi-angle laser light scattering experiment was performed using a Superdex 200 10/30 column (GE Healthcare) in continuous flow mode from the SEC column eluates using 1-s exposure periods for a total of 3600 s (one column volume). The data was integrated and reduced to produce the radially averaged scattering profiles of each individual frames [23,24].

For the structural studies, on-line size exclusion chromatography (SEC) was also employed as an additional purification method [25].

The molecular masses (MMs) were calculated from the SAXS data using the concentration-independent excluded Porod volume (MM_Porod_) [26]. The latter was determined given that the empirical ratio between the Porod volume (V_p_) and MM of a protein is approximately 1.65 [27]. A Bayesian inference approach was also applied yielding an accuracy above that of the individual methods, and reports MM (MM_Bayesian_) estimates together with a credibility interval [28].

Distance distribution function, p(r), and the maximum dimension of the scattering object, Dmax, were computed by the program GNOM [29] using the equation:

(1) $$p\left(r\right)=\frac{1}{2\pi^{2}}\int_{0}^{\infty }srI\left(s\right)sin\left(sr\right)ds$$

The low-resolution shapes of the full-length CBS-PPases were reconstructed ab initio from the p(r) function using a chain-like ensemble of dummy residues and the program GASBOR [30]. The program utilizes a simulated annealing algorithm to build models fitting the experimental data I_exp_(s) to minimize the discrepancy:

(2) $$\chi^{2}=\frac{1}{N-1}\underset{j}{\sum }{\left[\frac{I_{exp}\left(s_{j}\right)-cI_{calc}\left(s_{j}\right)}{\sigma \left(s_{j}\right)}\right]}^{2}$$

i.e., the reduced χ^2^ test, where N is the number of experimental points, c is a scaling factor and I_calc_(s_j_) and σ(s_j_) are the calculated intensity from the model and the experimental error of the momentum transfer s_j_, respectively. As an alternative to the reduced χ^2^ test, the Correlation Map method was also used to assess the quality of the model fits [31].

Hybrid rigid-body modeling was performed using the program CORAL [32], where the available high-resolution X-ray crystal structures of the PPase domains (PDB ID: 2haw, 3l31) were used. The program refines the relative positions and orientations of the high resolution models of the domains to build a composite model yielding the best fit to the experimental data. Theoretical scattering intensities from the atomic coordinates of the domains’ crystal structures were calculated with the program CRYSOL [33].

The GASBOR and CORAL outputs were analyzed using the programs SUPCOMB [34] and DAMAVER [35] to identify the most typical models best representing the spatial arrangement of the catalytic domain and the full-length CBS-PPases in solution.

The flexibility of the catalytic domain of dh-PPase (dh-PPaseΔCDC) and its probable conformations in solution were quantitatively assessed by the ensemble optimization method (EOM) [36]. This method selects an ensemble of possible conformers from a pool of randomly generated models, in this instance constructed from the available crystal structures of the domains of the homologous canonical PPase family II with a randomly generated linker region. CRYSOL was used to calculate the theoretical scattering from these models and a genetic algorithm was employed to select ensembles of conformations whose combined scattering profiles best fit the experimental data.

To analyze the amount of different conformations of dh-PPaseΔCDC solutions we used the program OLIGOMER [37]. Given the scattering intensities of components in a mixture, I_i_(s), the program fits the experimental scattering curve by their linear combination to determine their fractions w_i_. The equation:

(3) $$I\left(s\right)=\sum (w_{i}\times I_{i}\left(s\right))$$

is solved with respect to w_i_ by non-negative least-squares to minimize the discrepancy between the experimental and calculated scattering curves.

The ambiguity analysis of the obtained ab initio models was assessed by AMBIMETER [38]. The prediction of the protein shapes and their classification were carried out with DATCLASS [27].

## 3. Results

### 3.1. Structural Study of the Catalytical Domain of dh-PPase (dh-PPaseΔCDC) in Solution

The C-terminal domain of the canonical PPase of family II contains a high-affinity substrate-binding site, which changes its conformation from open to closed upon substrate binding [7]. The catalytic site binding the nucleophile-coordinating metal cations is located at the N-terminal domain. The N- and C-terminal domains are connected by a flexible linker (Gly188-Thr195). The canonical family II PPase was found to be dimeric in the crystal [8]. This PPase shows homology to the catalytic domain of the CBS-PPases, which comprises a common component of all specimens studied in this work. We have therefore first derived the structural model of CBS-PPase in solution utilizing the available crystal structures of the domains of the canonical PPase family II (PDB ID: 2haw and 1k23). To account for the flexibility of the linkers, EOM [36] was employed, which selects a sub-ensemble of conformations from a pool of models with randomly generated linkers to obtain the best fit to the experimental SAXS data. The flexibility analysis of the catalytic domain allowed us to choose the most probable domain conformation for further modeling of the full-length CBS-PPases from different bacterial species.

**Figure 1:**
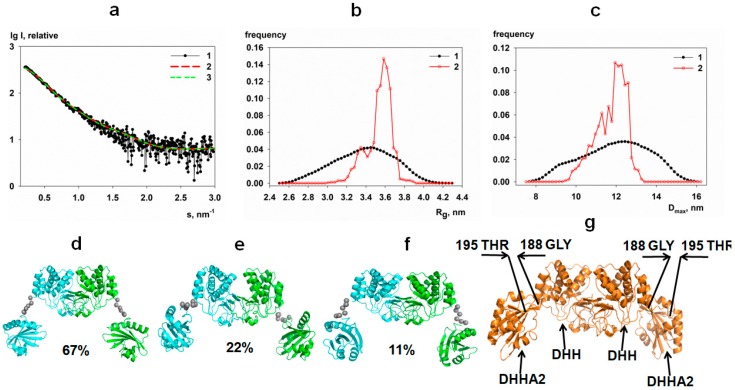
Small-angle X-ray scattering (SAXS) analysis of the catalytic domain of dh-PPase (dh-PPaseΔCDC): (**a**) Experimental data, curve 1; computed fit from the EOM selected ensemble of conformers, curve 2; OLIGOMER fit from the dh-PPaseΔCDC models displayed in (d-f), curve 3; (**b**, **c**) distribution histograms of R_g_ and D_max_ in the generated random pool (curve 1) and in the ensemble of the selected conformers (curve 2); (**d**–**f**) A set of the dh-PPaseΔCDC conformers with added flexible fragments (grey spheres) corresponding to the histogram peaks. The volume fraction of each species is indicated. (**g**) The crystallographic model of canonical PPase of family II (PDB ID: 1k23).

Figure 1 displays the experimental SAXS data from dh-PPaseΔCDC and the modeling results, revealing that the catalytic domain exists in solution as a homodimer with the conformation similar to that in the crystal. The sub-ensemble of conformations selected from a random pool of structures provides a good fit to the experimental data with χ^2^ = 1.06, CorMap 0.076 (Figure 1a, curve 2), while the fit from the crystallographic model of canonical PPase of family II (PDB ID: 1k23) yields a worse χ^2^ = 1.23 (Figure S2). The R_g_ and D_max_ distributions of the selected EOM ensemble are substantially narrower than those of the initial pool (Figure 1b,c). These results indicate that the catalytic domain of the CBS-PPase has a limited flexibility, and the dh-PPaseΔCDC homodimer is not completely extended. The most populated structures presented in Figure 1d–f are slightly different from each other, but all of them are in the open conformation. Their contributions to the experimental data was re-evaluated by the program OLIGOMER indicating that the model d in Figure 1 is the most populated (67 volume percent). Figure 1g demonstrates that the overall organization of dh-PPaseΔCDC is similar to that of the canonical PPase family II in open conformation, i.e., without substrate binding. Note that our SAXS measurements of the catalytic domain of CBS-PPase also were performed without substrate.

### 3.2. Structural Study of Full-Length CBS-PPases in Solution

Structural similarity of the catalytic domains of different PPases family II allows us to model a full-length structure of the CBS-PPase using the obtained dh-PPaseΔCDC model and available crystal structure of the regulatory domain (PDB ID: 3l31). As the CBS domains are known to promote aggregation of PPases in solution, on-line SEC-SAXS was employed to measure the scattering data from the full-length CBS-PPase constructs. The chromatogram peaks on the chromatograms (Figures S1–S3) clearly pointed to monodisperse species of all CBS-PPase solutions. Remarkably however, all SAXS-derived overall structural parameters of the full-length constructs as well as the hydrodynamic radii R_h_ (Table 1) were incompatible with dimeric constructs and indicated that the proteins exist as tetramers in solution.

The radii of gyration R_g_ calculated from the SEC-elution traces of CBS-PPases peaks (Figures S1 and S3) are in a good agreement with the values from the averaged data using Guiner approximation and also correlate well with the R_g_’s obtained from the p(r) function analysis (Table 1). The maximum sizes D_max_ predicted by DATCLASS [27] and obtained from p(r) function are also in good correlation.

The MMs determined from SEC-SAXS data and from the scattering curves based on the estimation of the Porod volume [26], MM_Porod_, and the Bayesian approach [28], MM_Bayesian_, correspond to the MMs of the tetramers calculated from amino acid sequence of the CBS-PPases (241 kDa for dh-PPase, 191 kDa for el-PPase, and 197 kDa for eh-PPase). These results clearly contradict the literature data [10], according to which the full-length proteins are homodimers.

### 3.3. Ab Initio Modeling

**Figure 2:**
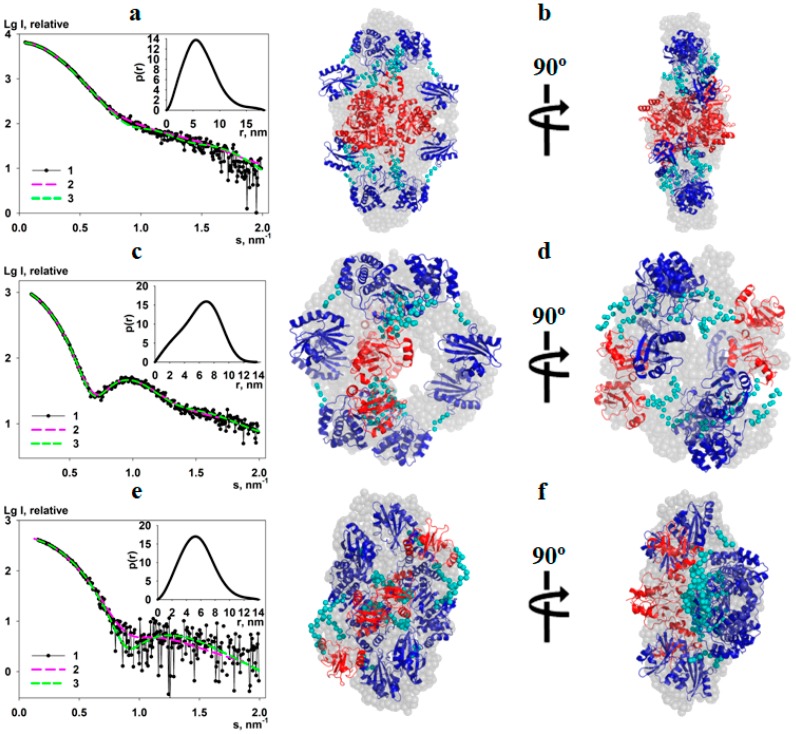
Scattering data from the full-length CBS-PPases and their shape restoration by ab initio and hybrid modeling methods. Panels (**a**,**c**,**e**) display the experimental SAXS data (1) and the fits from GASBOR (2) and CORAL (3) models; the distance distribution functions p(r) are displayed in the insets. Panels (**b**,**d**,**f**) present the comparison of ab initio GASBOR models (grey semitransparent spheres) and hybrid CORAL models (ribbons). From top to bottom: dh-PPase, eh-PPase, and el-PPase.

Low-resolution shapes of the proteins were generated ab initio by the program GASBOR [30]. The program uses dummy residues (DRs) as amino acids and employs simulated annealing to build a protein shape inside a sphere of size D_max_ (Figure 2a,c,e, inset). Since our data indicate that the proteins form a tetramer in solution P2 symmetry was applied. Typical ab initio shape reconstructions of the dh-PPase, eh-PPase and el-PPase presented in Figure 2b,d,f yield good fits to the experimental data (Figure 2a,c,e) with χ^2^ and CorMap of 1.19 and 0.003, respectively, for the dh-PPase, 1.23 and 0.001 for the eh-PPase, and 1.17 and 0.037 for el-PPase.

To assess the uniqueness of the ab initio models we used an a priori ambiguity measure based on the number of distinct shape topologies compatible with a given data set, which provide a quantitative ambiguity score. Generally, higher numbers of different topologies indicate higher probability of finding a false positive during the shape reconstruction. All obtained models have the ambiguity score 1.5 (Table 1) pointing to practically unique ab initio shape determination [38].

### 3.4. Hybrid Modeling

**Figure 3:**
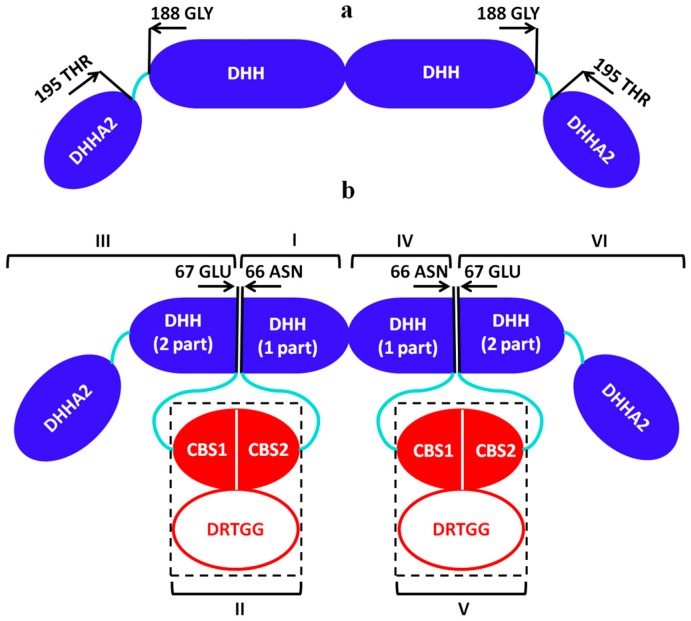
Schematic representation of the domains sequence of dh-PPaseΔCDC (**a**) and CBS-PPase dimer for the hybrid modeling (**b**). For dh-PPase, I, II (CBS+DRTGG domains), III, IV, and V (CBS+DRTGG domains) were used. For eh- and el-PPase, subunits I, II (only CBS domain), III, IV, V (only CBS domain) were used.

To obtain more detailed structural models of the full-length CBS-PPases and to better assess the relative positions of the catalytic and regulatory parts of CBS-PPases in solution, hybrid modeling was performed using the program CORAL [32]. Here, the model of the catalytic domain of CBS-PPase obtained above was utilized together with the available high-resolution X-ray crystal structure of the regulatory part (which consists of the CBS domains and an additional DRTGG domain in the case of dh-PPase (PDB ID: 3l31)). As the structures of the catalytic and regulatory domains are dimeric, each model was divided into two monomeric parts as illustrated in Figure 3.

In the primary sequence of the enzyme, the regulatory part is inserted in the DHH domain of the catalytic region between residues Asn66 and Gln67. Each monomer of the catalytic domain was thus divided into two parts: the first 66 amino acids of the DHH domain, DHH (part 1) and the rest of the catalytic part, DHH (part 2) + DHHA2. For the hybrid modeling we also used the two monomers of DHH (part 1) (denoted as I, IV in Figure 3), two monomers of DHH (part 2) domain together with DHHA2 (denoted as III, VI), two monomers of the CBS domains (denoted as II, V) and two monomers of CBS domain together with DRTGG domain (denoted as II, V) for dh-PPase. To reduce the number of free parameters and avoid data overfitting, the subunits I, III, IV and VI were grouped into one entity and the flexible links were reconstructed only between DHH and CBS domains as well as between DHH and DHHA2 domains. P2 symmetry was applied for the construction of the tetramer as a dimer of dimers. The obtained models presented in Figure 2b,d,f y fit the experimental data with χ^2^ = 1.62 (CorMap 0.00) for dh-PPase, χ^2^ = 1.13 (CorMap 0.075) for eh-PPase and χ^2^ = 1.03 (CorMap 0.084) for el-PPase (Figure 2a,c,e, curve 2).

As one can see, the models obtained by two independent methods agree well with each other (Figure 2), and this is corroborated by a quantitative assessment using a normalized spatial discrepancy (NSD, [34]), which yields the values of 1.74, 1.68 and 1.41 for dh-PPase, eh-PPase and el-PPase, respectively, when comparing GASBOR and CORAL models. Interestingly, the shapes of the rigid body models in Figure 2b,d,f also agree well with the shape and D_max_ predictions by the shape-classification tool DATCLASS [27] (Table 1). As predicted by DATSLASS, dh-PPase, and el-PPase are indeed rather compact, whereas eh-PPase forms a hollow structure in solution.

## 4. Discussion

Structural information on CBS-PPases is important for understanding the regulatory mechanism of the enzymes, in particular the signal transmission path from the regulatory to the active center. In the absence of high-resolution structural model of the full-length protein, the problem is difficult to tackle. The use of SAXS allowed us to construct a model revealing, for the first time, the spatial organization of the full-length CBS-PPases from three different species, *D. hafniense*, *E. lenta,* and *E. harbinense* in solution. We found that the three proteins form tetramers, all having the catalytic domain in an open conformation in the absence of the substrate, similarly to the canonical PPase family II [6]. Structural modelling with different approaches (ab initio and hybrid method) yielded consistent results and the ambiguity analysis [38] indicated that the models are likely to be unique further confirming the reliability of the results. Information on the oligomeric structures obtained by SAXS speaks in favor of a multilevel mechanism for the regulation of the CBS-PPases. The three enzymes studied by us were expressed from different bacterial species, but they all form tetramers in solution. The presence of the DRTGG domain in dh-PPase and its absence in el-PPase and eh-PPase suggests that this domain does not participate in the formation of the tetramer, while the regulatory insert promotes oligomerization [13,39].

Interestingly, the three proteins demonstrate different ways of tetramer formation: dh-PPase and el-PPase form rather compact tetramers, whereas eh-PPase adopts a hollow shape.

The significant change in the quaternary structure may be attributed to the difference in the primary sequence observed in both regulatory and catalytic parts of eh-PPase, i.e., in the most conserved regions of CBS-PPases. The primary sequence affects not only the overall shapes of the proteins, but also their functional properties. It has been shown earlier that Asn312 of the DHH domain is involved in kinetic cooperativity [40]. Its replacement by serine in dh-PPase led to the elimination of kinetic co-operativity in the enzyme and to the lack of kinetic cooperativity in eh-PPase, which contains a similar inherent mutation. Thus, the asparagine residue was considered to be indispensable in the cross-talk between the catalytic sites of the enzyme. Later bioinformatics analysis showed six polar amino acid residues of the dh-PPase as potentially important for the enzyme regulation [41]. It was found that three residues Arg295, Asn312, and Arg334 are crucial for CBS-PPase regulation via CBS domains. Their replacements by alanine abolished the kinetic cooperativity. Back replacement of serine with asparagine in eh-PPase partly restored kinetic cooperativity, providing additional support about the importance of asparagine for the cooperativity. Additionally, modeling and molecular dynamics simulations suggest destabilization of the subunit interface as a result of asparagine 312 and arginine 334 replacements by alanine, further emphasizing the importance of the structural organization of proteins for their functional properties [41].

The importance of the participation of the CBS domains in the oligomerization process is further emphasized by the involvement of these domains in signal transduction between the active and regulatory centers of CBS-PPases [42].

Generally, observed oligomerization of CBS-PPases can bring several functionally important advantages including thermodynamic stability and allosteric regulation of the enzymes [43,44,45]. In particular, due to oligomerization and formation of additional active or interactive sites in the interface regions between the CBS-PPase monomers, the affinity of the oligomers for substrates or binding partners may increase. Oligomerization is vital for the activity of many disease-related, e.g., viral, proteins, and the analysis of the oligomeric states has therefore direct therapeutic implications, especially taking into account that part of the family II PPases are hosted by human pathogens.

## 5. Conclusions

This study demonstrated, for the first time, that full-length wild-type CBS-PPases from three different bacterial species exist as stable tetramers in solution. The shapes of these tetramers are different but the oligomerization mechanisms appear to be similar and carried out through the CBS domains. The results of the present work may thus be important for further studies of the functional properties of these enzymes, and can serve as a structural basis for the understanding of the interaction between the individual protein domains and, therefore, for establishing the regulation mechanism of CBS-PPases.

## Figures and Tables

**Table 1:** Overall structural parameters of CBS-pyrophosphatases (CBS-PPases).

	dh-PPase	eh-PPase	el-PPase
***Guinier analysis***			
*R_g_* (nm)	4.95 ± 0.10	4.75 ± 0.38	4.29 ± 0.29
Molecular mass from Bayesian (credibility interval), kDa	243 (195–264)	186 (151–195)	208 (177–264)
***Analysis of the p(r) function***			
*R_g_* (nm)	4.94 ± 0.05	4.73 ± 0.05	4.24 ± 0.05
*D_max_*, (nm)	18.3 ± 1	13.7 ± 1	14.0 ± 1
*V_p_* (nm^3^)	412 ± 20	345 ± 17	368 ± 18
Molecular mass from Porod volume, kDa	250 ± 10	209 ± 10	223 ± 10
reciprocal-space fit to data (*χ*^2^, CorMap *P*)	1.09, 0.003	1.11, 0.01	1.18, 0.037
***Shape classification and ambiguity***			
Classification/(predicted *D_max_*, nm)	Compact (18.4)	Compact-hollow (14.4)	Compact (14.9)
Ambiguity score	1.176	0	0.30
Shape topologies	15	1	2
Uniqueness	Potentially unique	Potentially unique	Potentially unique
***Ab initio modelling***			
Method	GASBOR		
Symmetry imposed	P2	P2	P2
Model fits to data (*χ*^2^, CorMap *P*)	1.19, 0.003	1.23, 0.001	1.17, 0.037
***Atomistic modelling***			
Method	CORAL		
Symmetry imposed	P2	P2	P2
Model *R_g_* (nm)	4.93	4.80	4.23
Model fit to data (*χ*^2^, CorMap *P*)	1.62, 0.00	1.13, 0.075	1.03, 0.084
***MALLS-RI-UV MM and QELS R_h_*^1^**			
Calculated MM, amino acid sequence of monomer (kDa)	60.35	47.68	49.28
Average MM from MALLS/RI, kDa	254 ± 1	187 ± 1	190 ± 1
Hydrodynamic radius, R_h_ (nm)	5.62 ± 0.3	5.41 ± 0.2	4.37 ± 0.3

**^1^** Multi-Angle Laser Light Scattering (*MALLS*)—Refractive Index (*RI*)—Ultra Violet (*UV*) Molecular Mass (*MM*) and Quasi-Elastic Light Scattering (*QELS*) Hydrodynamic Radii (*R_h_*).

## Supplementary Materials

The following are available online at https://www.mdpi.com/2218-273X/10/4/564/s1, Figure S1: Comparison of experimental scattering data from full-length dh-PPase (**a**), eh-PPase (**b**) and el-PPase (**c**) at different concentrations 2, 5, 10 mg/mL (curves 1, 2 & 3, respectively), Figure S2: Scattering from the catalytic domain of dh-PPase (dh-PPaseΔCDC): 1, experimental data; 2, computed scattering from the crystallographic model of canonical PPase of family II (PDB ID: 1k23), Figure S3: The R_g_ and the integrated X-ray scattering intensities vs. data frame number through the SEC-SAXS traces of dh-PPase (**a**), eh-PPase (**b**) and el-PPase (**c**), Figure S4: The MM (orange) and differential reflective index (dRI) for the dh-PPase (**a**), eh-PPase (**b**) and el-PPase (**c**), Figure S5: The differential reflective index (dRI) and hydrodynamic radius (R_h_) correlation through the SEC-elution trace for the dh-PPase (**a**), eh-PPase (**b**) and el-PPase (**c**).
