# Supplementary material for: Tetrameric Structures of Inorganic CBS-Pyrophosphatases from Various Bacterial Species Revealed by Small-Angle X-ray Scattering in Solution
Source: Biomolecules. 2020 Apr 7;10(4):564. doi: 10.3390/biom10040564 (PMC7226116; doi:10.3390/biom10040564)
Supplement: Supplementary file 1 [file biomolecules-10-00564-s001.pdf]

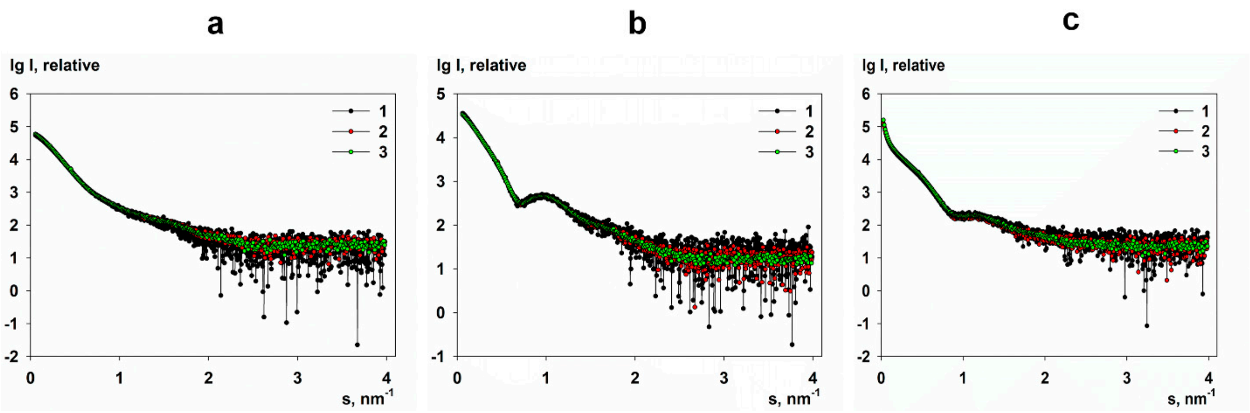

Figure S1. Comparison of experimental scattering data from full-length dh-PPase (**a**), eh-PPase (**b**) and el-PPase (**c**) at different concentrations 2, 5, 10 mg/mL (curves 1, 2 & 3, respectively).

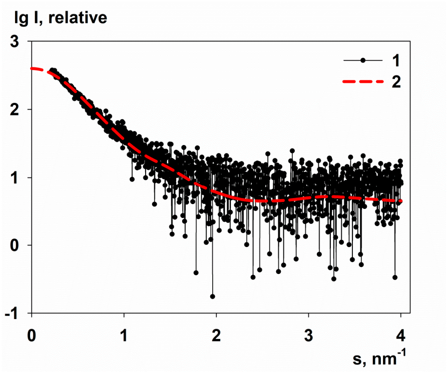

Figure 2. Scattering from the catalytic domain of dh-PPase (dh-PPaseΔCDC): 1, experimental data; 2, computed scattering from the crystallographic model of canonical PPase of family II (PDB ID: 1k23).

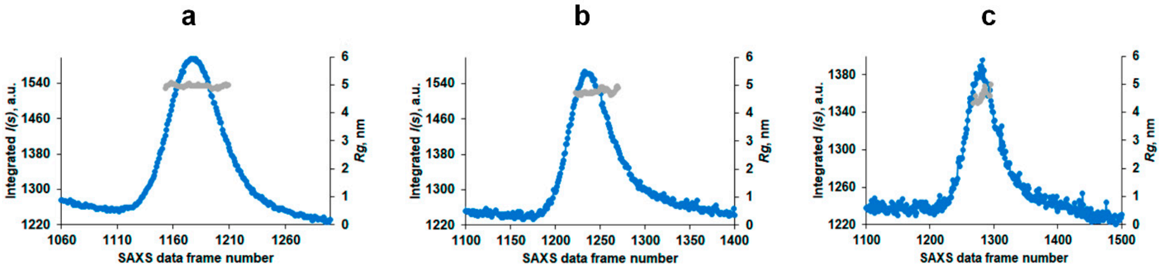

Figure 3. The R_g and the integrated X-ray scattering intensities vs. data frame number through the SEC-SAXS traces of dh-PPase (**a**), eh-PPase (**b**) and el-PPase (**c**).

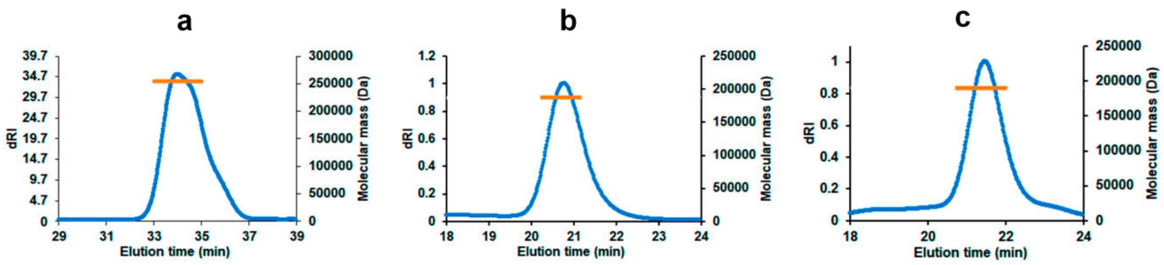

Figure 4. The MM (orange) and differential reflective index (dRI) for the dh-PPase (**a**), eh-PPase (**b**) and el-PPase (**c**).

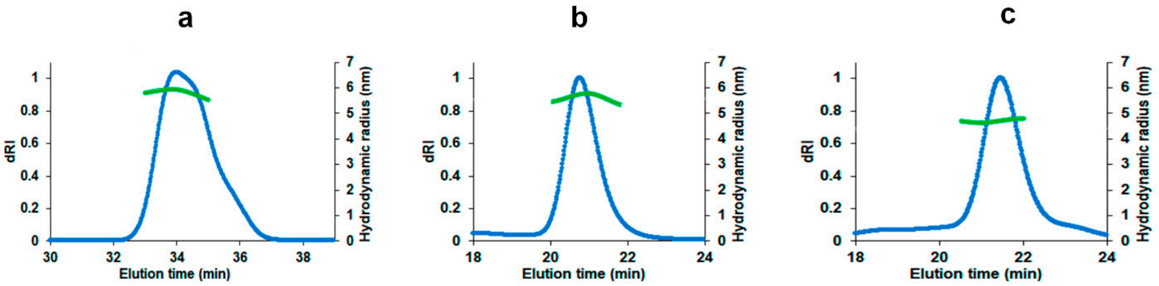

Figure 5. The differential reflective index (dRI) and hydrodynamic radius (R_h) correlation through the SEC-elution trace for the dh-PPase (**a**), eh-PPase (**b**) and el-PPase (**c**).
